# The relationship between stability of interpersonal coordination and inter-brain EEG synchronization during anti-phase tapping

**DOI:** 10.1038/s41598-022-10049-7

**Published:** 2022-04-13

**Authors:** Yuto Kurihara, Toru Takahashi, Rieko Osu

**Affiliations:** 1 Graduate School of Human Sciences, Waseda University, 2-579-15 Mikajima, Tokorozawa, Saitama Japan; 2 Faculty of Human Sciences, Waseda University, 2-579-15 Mikajima, Tokorozawa, Saitama Japan

**Keywords:** Neuroscience, Psychology

## Abstract

Inter-brain synchronization is enhanced when individuals perform rhythmic interpersonal coordination tasks, such as playing instruments in music ensembles. Experimentally, synchronization has been shown to correlate with the performance of joint tapping tasks. However, it is unclear whether inter-brain synchronization is related to the stability of interpersonal coordination represented as the standard deviation of relative phase (SDRP). In this study, we simultaneously recorded electroencephalograms of two paired individuals during anti-phase tapping in three interactive tapping conditions: slow (reference inter-tap interval [ITI]: 0.5 s), fast (reference ITI: 0.25 s), and free (preferred ITI), and pseudo tapping where each participant tapped according to the metronome sounds without interaction. We calculated the inter-brain synchronization between pairs of six regions of interest (ROI): frontal, central, left/right temporal, parietal, and occipital regions. During the fast tapping, the inter-brain synchronization significantly increased in multiple ROI pairs including temporoparietal junction in comparison to pseudo tapping. Synchronization between the central and left-temporal regions was positively correlated with SDRP in the theta in the fast condition. These results demonstrate that inter-brain synchronization occurs when task requirements are high and increases with the instability of the coordination.

## Introduction

People interact during group dancing and music ensembles by coordinating their actions swiftly and accurately^1^. These widespread social activities involve temporally precise interpersonal synchronization based on the information exchanged via multiple sensory modalities^2^. Furthermore, these social activities require that individuals coordinate stably to exhibit their performance^3,4^. Previous studies have examined the stability of interpersonal coordination using simple joint tapping tasks, such as in-phase or anti-phase tapping between two individuals^4–6^.

Interpersonal coordination patterns can be represented by a relative phase (RP) that captures the angular differences between two oscillators^7–10^. The standard deviation of the relative phase (SDRP) represents the instability of the coordination patterns. Two patterns of interpersonal coordination have been examined, in-phase (RP = 0°) and anti-phase (RP = 180°) modes^11^. In-phase coordination is more stable than anti-phase coordination^7,8^. In particular, the anti-phase interpersonal coordination becomes increasingly unstable (increase in SDRP) as the movement frequency increases, eventually breaking down and transiting to in-phase coordination (generally called phase transition)^7,12^. For instance, Schmidt et al. observed that, when two participants coordinated leg movements with one another, the SDRP for the anti-phase mode was larger than that for the in-phase mode, and transition from the anti-phase to in-phase coordination was noted when the frequency of leg movement increased^7^.

To elucidate the neural mechanisms of interpersonal coordination, hyperscanning has been used to examine the synchronization of two or more brains (inter-brain synchronization)^13,14^ during a variety of interaction tasks from simple joint tapping tasks^15,16^ to complex natural tasks, such as conversations^17^. Previous research demonstrates a relationship between inter-brain synchronization of electroencephalogram (EEG) signals and behavioral performance, that is, higher synchronization indicates better achievement in an interpersonal coordination task^18,19^. For instance, Kawasaki et al. suggested that good performance pairs of visually guided alternate tapping showed higher inter-brain EEG synchronization in the alpha frequency (12 Hz) than poor performance pairs^15^. These previous hyperscanning studies focused on behavioral performance representing the degree of accomplishment of the task required by the experimenter. However, none have examined the relationship between inter-brain synchronization and the stability of interpersonal coordination. If stability reflects performance and performance correlates with inter-brain EEG synchronization, inter-brain EEG synchronization would be higher when the interaction is more stable.

In this study, to elucidate the relationship between the stability of interpersonal coordination and inter-brain synchronization, we examined the SDRP and inter-brain EEG synchronization during anti-phase finger tapping, which is less stable than in-phase tapping, especially when the tapping speed is increased^7^.

**Figure 1:**
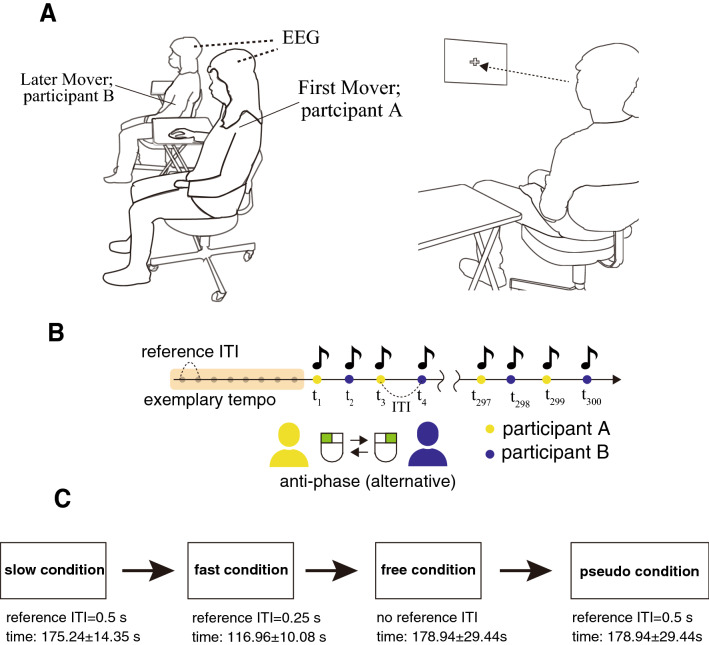
Experimental setting and the procedure of anti-phase tapping tasks. (**A**) We conducted hyperscanning using two wireless EEG systems. Each participant gazed at a fixation point in front of him/her during anti-phase tapping. (**B**) The anti-phase tapping tasks consisted of slow, fast, and free speed conditions. In the slow and fast conditions, the participants first listened to exemplary sounds (reference ITI) of a slow (0.5 s) and a fast (0.25 s) frequency. After the participants listened to the sound, they started to perform anti-phase tapping with the same frequency as that of the reference ITI. In the free condition, there was no reference sound. Thus, the participants performed the tapping with preferred frequency in the free condition. (**C**) The figure indicates the flow of the experiments. First, participants performed three interactive tapping conditions: slow, fast, and free, in that order. Then, they performed pseudo tapping conditions.

Nineteen pairs of participants performed anti-phase interactive finger tapping in slow (requested inter-tap interval [ITI]: 0.5 s), fast (requested ITI: 0.25 s), and free speed conditions (preferred ITI) by hearing the sounds of his and partner’s taps. The tempo was indicated by eight beeps before starting to tap (Fig. 1). Participants then performed a control condition of pseudo tapping where each participant tapped to the metronome sounds without coordinating with each other, during which 29 channel EEG was measured. We focused on the theta (4–7 Hz), alpha (8–12 Hz), and beta (13–30 Hz) bands of inter-brain synchronization for which relevance to interactive behavioral performance has been demonstrated in previous hyperscanning studies^19–22^ and which are less susceptive to artifacts caused by body motion or EMG. We computed circular correlation coefficient (CCorr) and Phase Locking Value (PLV) for each region of interest (ROI) pair as an index of inter-brain synchronization and examined correlation with SDRP of finger tapping.

The remainder of the paper is organized as follows: in the “Results”, we first present the behavioral measures of anti-phase tapping. Next, we compare inter-brain EEG synchronization during interactive tapping to that of pseudo tapping without interaction. Then we examine the correlation between SDRP and inter-brain EEG synchronization. In the “Discussion”, we present the study findings in terms of the extant literature and the study limitations. In the “Methods”, we describe the details of experimental procedures and analyses.

## Results

### Behavioral measurements during anti-phase tapping

**Figure 2:**
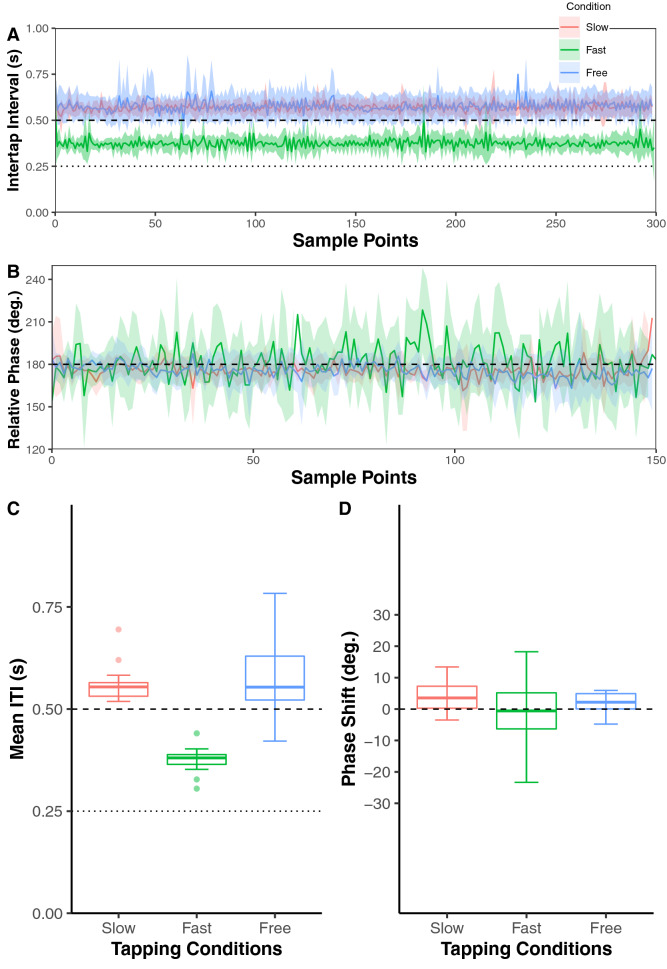
The behavioral analysis of anti-phase tapping in slow, fast, and free tapping condition. (**A**) The curves show the ensemble average of inter-tap interval (ITI) time series across participants. The light color areas indicate the standard error of ITI. The dashed line indicates the reference ITI in the slow condition. The dotted line indicates the reference ITI in the fast condition. (**B**) The curves show the ensemble average of the relative phase (RP) time series across participants. The light color areas indicate the standard error of RP. The dashed line indicates the anti-phase angle. (**C**) The box plots show the median and interquartile range (IQR) of the mean inter-tap intervals (mean ITI). (**D**) The box plots show the median and IQR of the phase shift of tapping.

We calculated the inter-tap interval (ITI)^5,6,23^ by subtracting the tap onset time of one participant from the subsequent tap onset time of the other to see whether participants performed anti-phase tapping to the reference ITI (Fig. 1). Figure 2A,B depict ensemble-averaged time series of ITI and relative phase (RP) and their standard deviation (SD). In order to confirm that averaged ITI and RP time series were stationary processes, we performed Dickey-Fuller tests^24^. The averaged ITI and RP time series were confirmed as stationary processes in the slow, fast, free, and pseudo conditions.

We calculated the average of ITI (mean ITI), phase shift (shift from 180° computed by subtracting the RP from 180°), and SDRP (standard deviation of RP). The average and SD of the mean ITI in the different conditions were as follows: slow (0.565 ± 0.047 s), fast (0.374 ± 0.033 s), and free (0.577 ± 0.10 s). Thus, we confirmed that the mean ITI was longer than the reference ITI in slow and fast conditions. In addition, we calculated phase shift to confirm the shifting from the reference RP (180°) in the slow, fast, and free conditions (Fig. 2D). The mean and SD of absolute phase shift was 4.911° and 3.944° in the slow condition, 13.80° and 17.30° in the fast condition, and 5.945° and 11.87° in the free condition, respectively.

**Figure 3:**
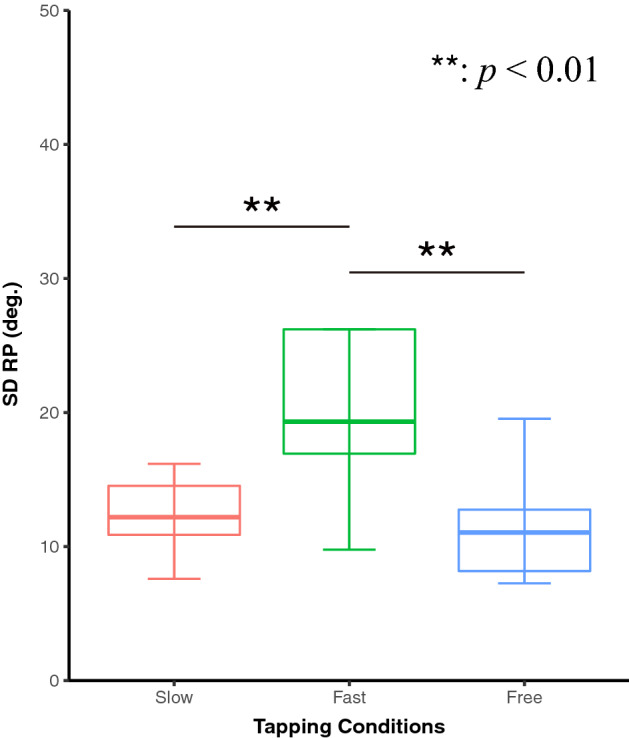
The difference of SDRP among slow, fast, and free tapping conditions. The box plots show the median and interquartile range (IQR) of SDRP. The SDRP in fast conditions was significantly larger than in slow and free conditions.

The one-way repeated measures analysis of variance (ANOVA) was conducted to compare SDRPs among slow, fast, and free conditions (Fig. 3). To confirm the equality of variances of the differences among levels, sphericity was examined by Mauchly’s test^25^. The degree of freedom was not corrected because the assumption of sphericity was not violated (*p* = 0.064). ANOVA with tapping conditions revealed the main effects of tapping conditions on SDRP (*F*_2,24_ = 8.928, *p* = 0.001*, η*_*p*_^*2*^ = 0.427; Fig. 3). Post-hoc paired t-tests using a Holm correction confirmed that the SDRP of the fast condition was larger than those of slow and free conditions (slow vs. fast, *t*_*12*_ =  − 3.73, *p*_*adj*_ = 0.003*, d* =  − 1.036; fast vs. free, *t*_*12*_ = 3.580, *p*_*adj*_ = 0.003*, d* = 0.993; and slow vs. free, *t*_12_ =  *− *0.154, *p*_*adj*_ = 0.879, *d* =  *− *0.043). Thus, participants performed the anti-phase tapping with high variability in the fast condition.

### Inter-brain EEG synchronization during anti-phase interactive tapping in comparison to that during pseudo tapping

**Figure 4:**
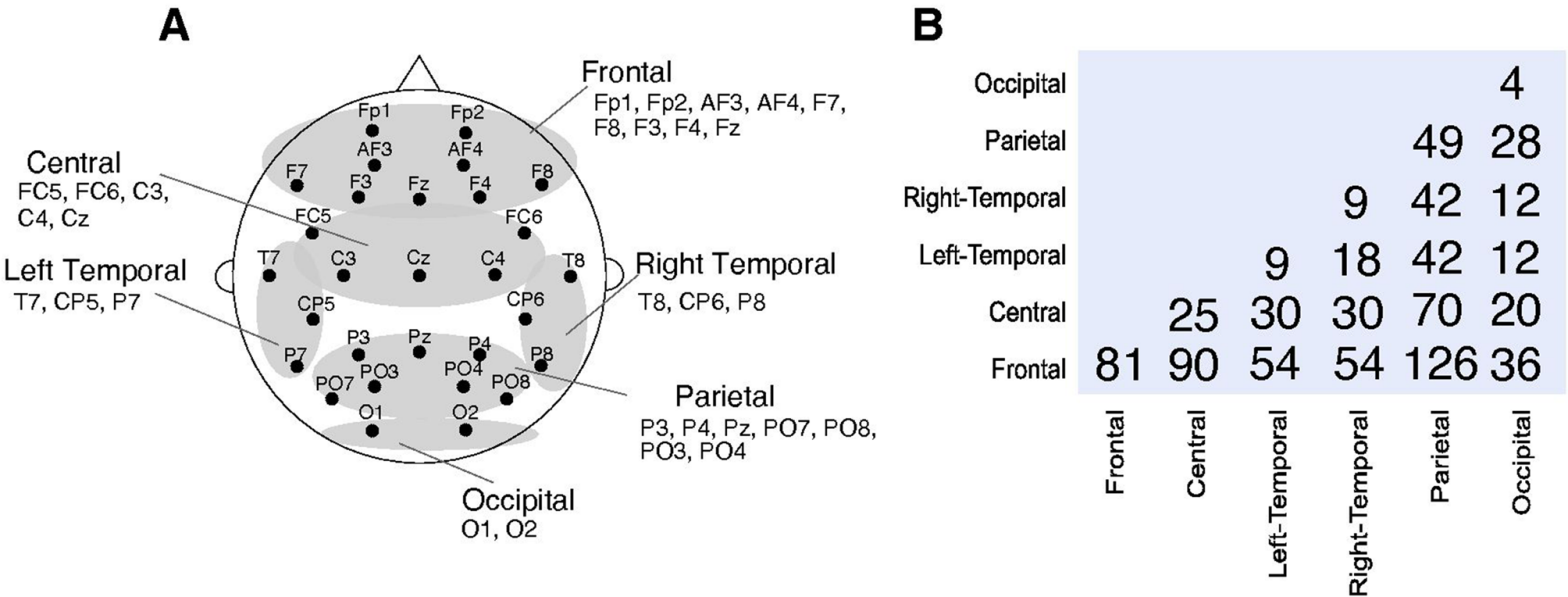
Six regions of interest and the number of channel pairs included in each ROI pairs. (**A**)The electrodes were classified into six regions of interest: frontal (Fp1, Fp2, AF3, AF4, F7, F8, F3, F4, and Fz), central (FC5, FC6, C3, C4, and Cz), left temporal (T7, CP5, and P7), right temporal (T8, CP6, and P8), parietal (P3, P4, Pz, PO7, PO8, PO3, and PO4), and occipital (O1, and O2) areas. (**B**) The matrix shows the number of inter-brain channel pairs between which CCorr and PLV were computed for each ROI pairs.

The inter-brain EEG synchronization was evaluated using CCorr^26,27^ and PLV^28^ for each electrode pair between two participants in theta, alpha, and beta frequency bands. In addition, we calculated inter-brain synchronization in low beta (13–16 Hz; beta1), middle beta (16.5–20 Hz; beta2), and high beta (20.5–28 Hz; beta3) frequency bands (see Section 3 of Supplementary Information). Because each participant had 29 electrodes, there were 841 combinations of electrode pairs. The calculated CCorr was normalized by Fisher’s Z transformation. We categorized the 29 electrodes into six regions of interest (ROIs): frontal (Fp1, Fp2, AF3, AF4, F7, F8, F3, F4, and Fz), central (FC5, FC6, C3, C4, and Cz), left temporal (T7, CP5, and P7), right temporal (T8, CP6, and P8), parietal (P3, P4, Pz, PO7, PO8, PO3, and PO4), and occipital (O1 and O2)^17,29,30^ (Fig. 4) from EEG power topo spectra topographical map and significant inter-brain connections (see Sections 4 and 5 of Supplementary Information), resulting in 21 ROI pairs (see “Methods” for detail). Then, we averaged the CCorrs or PLVs of electrode pairs within each ROI pair.

To examine if the observed EEG synchronization was coincidentally obtained or not, the difference in ROI-averaged CCorr/PLV between interactive tapping and pseudo tapping was tested by one-tail Wilcoxon signed rank test^31^ with false discovery rate (FDR) correction (number of comparisons was 21 ROI pairs) for each tapping condition (slow, fast, and free) and frequency band.

**Figure 5:**
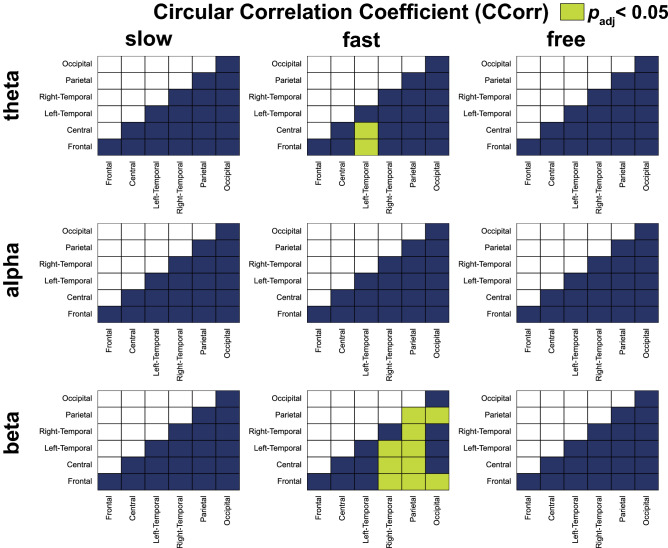
Comparison between CCorr of pseudo condition and CCorr of slow, fast, and free conditions in theta, alpha, and beta frequency bands. The matrices contain the p-value of Wilcoxon signed rank test comparing CCorrs during interactive tapping (slow, fast, and free conditions) and pseudo tapping in each pair of regions of interest. ROI pairs that had significantly larger CCorrs between participants during anti-phase tapping compared to pseudo tapping are indicated by light green in the corresponding cells with *p*_*adj*_ < 0.05.

Figure 5 shows the ROI pairs that had significantly larger CCorrs in each condition and each frequency band in comparison to the pseudo tapping condition. For the theta band, we observed significantly larger CCorrs in two ROI pairs in the fast condition. For the alpha band, there were no significant CCorrs in the ROI pairs in the fast condition. For the beta band, we detected significantly larger CCorrs in the 10 ROI pairs in the fast condition.

**Figure 6:**
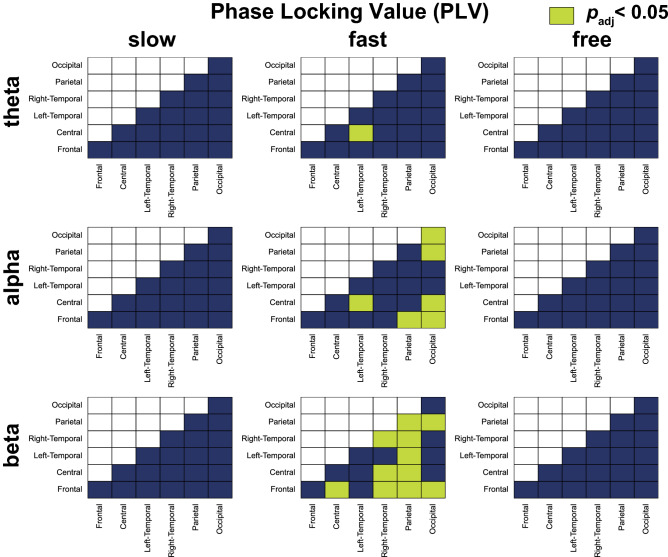
Comparison between PLV of pseudo condition and PLV of slow, fast, and free conditions in theta, alpha, and beta frequency bands. The matrices contain the p-value of Wilcoxon signed rank test comparing PLVs during interactive tapping (slow, fast, and free conditions) and pseudo tapping in each pair of regions of interest. ROI pairs that had significantly larger PLVs between participants during anti-phase tapping compared to pseudo tapping are indicated by light green in the corresponding cells with *p*_*adj*_ < 0.05.

Figure 6 shows the ROI pairs with significantly larger PLVs in each condition and each frequency band in comparison to the pseudo tapping condition. As for the theta band, we found significantly larger PLVs in the one ROI pairs in the fast condition. For the alpha band, significantly larger PLVs were observed in six ROI pairs in the fast condition. For the beta band, we found significantly larger PLVs in the 11 ROI pairs in the fast condition.

We additionally conducted a comparison with surrogate data generated by temporal shuffling to verify that the results were robust against to a differences of control methods (see Section 2 of Supplementary Information). We obtained significant inter-brain EEG synchronization in multiple ROI pairs in the fast condition, which replicates the results using pseudo tapping as a control.

### Relationship between inter-brain EEG synchronization and the stability of anti-phase tapping

**Figure 7:**
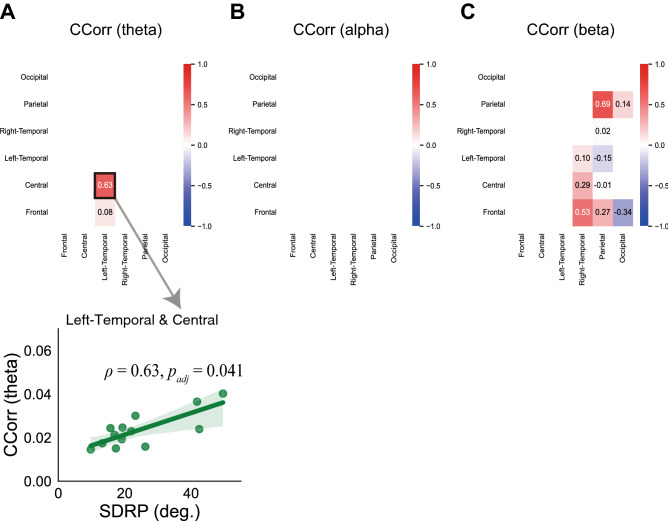
Relationship between inter-brain CCorr and the instability of anti-phase tapping in the fast condition. The heatmap indicates Spearman’s correlations between SDRP and CCorr in the theta (**A**), alpha (**B**), and beta (**C**) frequency bands. The scatter plot indicating significant correlations is shown.

We calculated the Spearman correlation between SDRP and ROI-averaged CCorrs that were significantly larger than pseudo tapping in the fast condition. In the theta frequency bands, the SDRP was significantly correlated with the CCorr of the left temporal and central ROI pair (*ρ* = 0.63, *p*_*adj*_ = 0.041; Fig. 7A). However, no ROI pair showed significant correlations between the SDRP and CCorrs in the alpha or beta bands (Fig. 7B,C).

**Figure 8:**
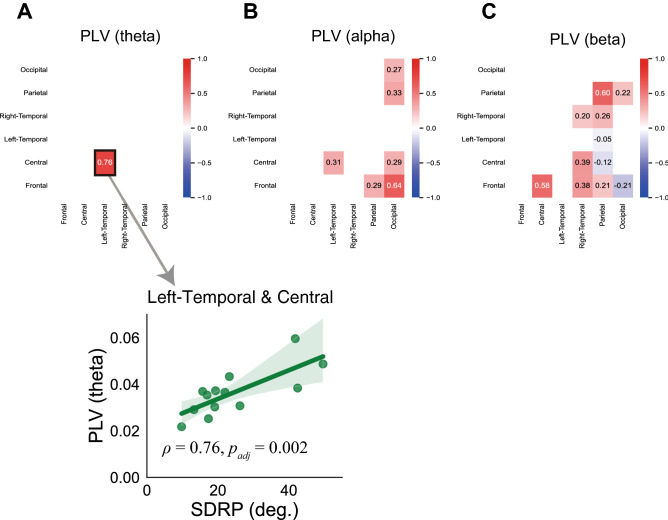
Relationship between inter-brain PLV and the instability of anti-phase tapping in the fast condition. The heatmap indicates Spearman’s correlations between SDRP and CCorr in the theta (**A**), alpha (**B**), and beta (**C**) frequency bands. The scatter plot indicating significant correlations is shown.

We calculated the Spearman correlation between SDRP and ROI-averaged PLVs that were significantly larger than pseudo tapping in the fast condition. In the theta frequency bands, the SDRP was significantly correlated with the PLV of the left temporal and central ROI pair (*ρ* = 0.75, *p*_*adj*_ = 0.013; Fig. 8A). However, no ROI pair showed significant correlations between the SDRP and PLVs in the alpha or beta bands (Fig. 8B,C).

In general, instability (SDRP) correlated with tapping speeds^7,12^. To test if the observed correlation was not due tapping speed but instability, we tested if the mean inter tap interval (meanITI) correlate with ROI-averaged CCorr/PLV for the fast condition for each frequency band, and found no significant correlation.

## Discussion

This study examined the relationship between the behavioral stability of interpersonal coordination and inter-brain EEG synchronization. Participants performed anti-phase tapping in three different speed conditions. We observed significantly high inter-brain EEG synchronization in multiple ROI pairs in the theta, alpha, and beta bands during anti-phase interactive tapping with fast speed as compared to the pseudo tapping. The two brains did not synchronize when tapping speed was slow. Among these synchronized ROI pairs, tapping instability (SDRP) and inter-brain synchronization, measured as both CCorr and PLV, were positively correlated between the left temporal region of one participant and the central regions of the other paired participant in the theta frequency bands. Since neither CCorr nor PLV of this ROI pair correlated with tapping speed, the left temporal-central inter-brain synchronization seems to be relevant to instability rather than speed.

In this study, we focused on the SDRP of anti-phase tapping. The anti-phase tapping spontaneously transited to in-phase tapping as the coordination was unstable^7,12^. SDRP of anti-phase tapping can be regarded as an indicator of how well the participant maintains a constant anti-phase tapping tempo. Our results show that with worsened anti-phase tapping performance (high SDRP), inter-brain synchronization increases between the central and left temporal regions in theta band. These results appear to be in opposition to those of Kawasaki et al., who proposed that high inter-brain synchronization is related to good performance^16^. However, because the performance indices differ between the studies it is not possible to directly compare them. Kawasaki et al. regarded good performance when the difference in interval between two consecutive taps was < 50 ms, and assessed the binary expression (i.e., good/poor), while SDRP was a continuous variable. There was also a difference in degrees of difficulty, as Kawasaki et al. conducted the tapping task at a preferred speed (~ 0.50 s) that was equivalent to the slow and free conditions in our study where we failed to detect a significant inter-brain synchronization. There is a possibility that the increase in inter-brain EEG synchronization was caused by increasing tapping speed rather than increasing instability because the anti-phase interpersonal coordination becomes increasingly unstable as the movement frequency increases. By limiting the correlation analysis to the data in the fast condition, we were able to find the ROI synchronizations, irrespective of movement speed.

We observed a significant inter-brain EEG synchronization of theta (4–7 Hz), alpha (8–12 Hz), and beta (13–30 Hz) frequency bands in CCorr and PLV with the fast tapping. We observed inter-brain beta EEG synchronization in larger area than other frequency bands. Previous studies have shown that beta synchronization reflects the neural correlation of higher-level interactions^21,32^. Therefore, the observed beta synchronization of the two brains might be ascribed to the reflection of high cognitive loading in fast anti-phase tapping because it is difficult to keep tapping stationary between two participants. However, in fact, it is not yet clear which frequency band is significantly related to interpersonal coordination^33^. Shiraishi et al. found the EEG theta (4–7 Hz) oscillations were more synchronized during turn-taking cooperative tapping actions^22^. Yun et al. observed a significant increase in the inter-brain EEG synchronization in theta and beta frequency bands during unintentional body movements^21^. Kawasaki et al. showed that the inter-brain EEG synchronization in the alpha frequency (about 12 Hz) was higher in good tapping pairs than that in poor tapping pairs^16^. In the interpersonal coordination task of finger tapping, stimulating the left M1 of the pairs on beta rhythm (20 Hz) using transcranial alternating current stimulation (tACS) enhanced tapping performance^34^. No previous hyperscanning studies have conclusively shown which frequency band is essential to interpersonal coordination, which needs to be addressed in future studies.

As indices for inter-brain synchronization, we computed both CCorr and PLV. In the field of EEG Hyperscanning, Phase Locking Value (PLV) is the most commonly used index of inter-brain EEG synchronization. Circular Correlation Coefficient (CCorr) has recently attracted attention after Burgess demonstrated its advantage over PLV or coherence^25^ in the sense that it is robust against coincidental synchronization. However, there is not yet a consensus of what measure is optimal for EEG hyperscanning studies. PLV and CCorr are similar in the sense that it they focus on phase synchrony. Coherence is also used in some hyperscanning studies^35^, but coherence includes the amplitude information in addition to phase synchrony. In the present study, we obtained similar results in CCorr and PLV but different ones in Coherence (see Section 1 of Supplementary Information).

Our results reveal the possibility that the central and left-temporal inter-brain EEG synchronization might be related to the instability of interpersonal coordination. The observed correlation may be ascribed to auditory-motor coordination. In a joint tapping task for which feedback is a visual image, inter-brain EEG synchronizations in the central (i.e., motor area) and posterior area (i.e., visual area) were related to the tapping performance^16^. In our experiment, anti-phase tapping used sound feedback. Temporal regions respond to sounds^36^ and central regions respond to tapping coordination^37^. Auditory-motor coordination, similarly to visuo-motor coordination, might have caused the inter-brain synchronization between the central and temporal regions. To identify the area more accurately, EEG source estimation using individual MRI structures is preferable, which is one of our future directions.

In the fast condition, we observed the inter-brain beta EEG synchronization in multiple left temporal and parietal ROI pairs (Figs. 5, 6) including temporoparietal junction (TPJ). Many imaging studies with a single brain have revealed that bilateral TPJ is specifically involved in reasoning regarding the contents of another person’s mind, i.e., "theory of mind"^38–40^ using tasks such as reading social stories. An EEG study by Park et al. also indicated that beta oscillations of the right TPJ are related to the reasoning of other’s preference^41^. Recent hyperscanning studies also observed inter-brain synchronization including left TPJ. For example, Jiang et al. observed high inter-brain synchronization for leader–follower in the left TPJ during verbal communication using fNIRS hyperscanning^42^. Abe et al. reported that the right TPJ is activated during joint action using fMRI hyperscanning^43^. In our fast tapping task, for successful anti-phase tapping, participants had to estimate their partner's ability to keep tapping quickly or preference for tapping tempo, which might have induced the inter-brain synchronization of social areas including the TPJ.

Our study has the following limitations. First, the small sample size limited the statistical power of the study. We compared stranger to acquaintance pairs in the tapping behavior and inter-brain EEG synchronization; however, no significant differences were observed. Future studies with larger samples sizes will clarify the interpersonal relationships and inter-brain synchronization in more detail. Second, the classification of EEG channels into ROIs used in this study is not necessarily a standardized method. Future studies should conduct EEG source estimation using individual MRI structures with more EEG channels. Third, although we detected a correlation between the instability of interpersonal coordination and inter-brain synchronization, the causality remains unclear. The modulation of inter-brain synchronization using tACS^44^ would reveal the causality between interpersonal coordination and inter-brain synchronization.

In summary, our findings support the hypothesis that inter-brain EEG synchronization is related to the stability of interpersonal coordination. However, our results revealed that the correlation is negative, i.e., inter-brain synchronization increased as the behavior became more unstable in the central and left-temporal regions in theta bands. It is possible that high inter-brain synchronization promotes cooperation to maintain the stability of interpersonal coordination.

## Methods

### Ethics

The experimental procedures were approved by the Ethical Review Committee of Waseda University. All experiments were performed in accordance with relevant guidelines and regulations. All participants provided written informed consent.

### Participants

Nineteen pairs of right-handed participants (8 male pairs, 11 female pairs; mean age = 22.5 years, SD = 4.3) were enrolled. Two male pairs and four female pairs were excluded because of recording artifacts: two female pairs because of device trouble and the others because of EEG artifacts. Thus, 13 pairs (eight acquaintance and five stranger pairs) were included.

### Experimental settings and behavioral task

The participants in each pair were seated side-by-side (Fig. 1A). They were asked to gaze at a fixation point during the tapping task to avoid possible synchronization resulting from mutual glances. The distance between the participants was approximately 70 cm. The participants were asked to perform anti-phase tapping tasks using two computer mice. These computer mice were placed on two tables (40 × 50 cm). The participant who tapped first used the mouse’s left click with his/her right index finger. The other participant used the right click of the mouse with his/her right index finger. Tapping produced sound feedback (sound frequency, 440 Hz). Each participant listened to the sound produced by his/her own tap and his/her partner’s tap through earphones. The anti-phase mode is beneficial in avoiding the spurious inter-brain EEG synchronization caused by the movement similarity across individuals compared to in-phase tapping^44^.

The experiment included three interactive tapping conditions that consisted of different tapping frequencies: slow condition (reference ITI = 0.50 s), fast condition (reference ITI = 0.25 s), free condition (an ITI preferred by the pair), and one pseudo tapping condition without interaction (tapping according to 0.50 s metronome sounds). In the slow and fast conditions, the participants first listened to the exemplary frequency (Fig. 1B). After the first eight sounds were transmitted, the metronome was switched off, and the pairs started tapping at a frequency as close as possible to the memorized reference frequency. In the free condition, there was no reference frequency; the participants tapped at a preferred frequency. In the pseudo condition, after eight sounds of 2 Hz, the participants continued tapping to the 2 Hz metronome independently with each other. There was no sound feedback of taps for the participants in the pseudo condition. A trial in each condition was completed after 300 taps. Therefore, the slow, fast, free, and pseudo conditions had different durations for one trial (slow: mean, 175.24 s, SD, 14.35 s; fast: mean, 116.96 s, SD, 10.08 s; free: mean, 178.94 s, SD, 29.44 s; pseudo: mean, 160.28 s, SD, 0.294 s). All conditions consisted of only one trial. The order of the first and second mover who started tapping was fixed across all conditions.

### Behavioral analysis

We calculated the ITI by subtracting the tap onset time from the adjacent tap onset time. ITI was defined using tapping times as follows:

1 $$\begin{array}{c}{ITI}_{n}=t\left(n+1\right)-t\left(n\right)\end{array}$$

2 $$\begin{array}{c}Mean \; ITI=\frac{1}{N}\sum_{k=1}^{N}{ITI}_{k}\end{array}$$

where t(n) denotes the n-th tap timing, N is the number of tapping, mean ITI indicates the average of ITI. We calculated the RP of tapping using a circular measure to confirm whether tapping was anti-phase (RP = 180°)^9,34^. RP was defined using tapping times:

3 $$\begin{array}{c}RP=\left[\frac{T(n{)}_{pB}-T(n{)}_{pA}}{\left.T(n+1{)}_{pA}-T(n{)}_{pA}\right)}\right]\times 360\end{array}$$

4 $$\begin{array}{c}Phase \; Shift=180-arg\left\{\sum_{k=1}^{N}\mathrm{exp}\left(i {RP}_{k}\right)\right\}\end{array}$$

where $T(n{)}_{pA}$ and $T(n{)}_{pB}$ denote the tapping time (second) of participants A and B, respectively. The RP ranged from 0° to 360°. The phase shift from anti-phase mode (180°) was calculated by subtracting average RP from 180°. The $i$ shows the complex number and the $\mathrm{arg}\{\bullet \}$ shows the argument of complexity. We calculated the circular standard deviation of RP (SDRP) as follows:

5 $$SDRP=\sqrt{-2\mathrm{log}R}$$

6 $$R=\left|\frac{1}{N}\sum_{k=1}^{N}\mathrm{exp}\left(i {RP}_{k}\right)\right|$$

where SDRP represents the instability of anti-phase tapping. R is the resultant length of the mean RP vector.

### EEG hyperscanning data recording and analyses

The brain activities of each pair were simultaneously recorded by two EEG systems, each with 29 active scalp electrodes (Quick-30; Cognionics, San Diego, CA, USA) in accordance with the placement of the international 10/20 system: Fp1/Fp2, AF3/AF4, F3/F4, F7/F8, FC5/FC6, C3/C4, T7/T8, CP5/CP6, P3/P4, P7/P8, PO3/PO4, PO7/PO8, O1/O2, Fz, Cz, and Pz. The sampling rate was 500 Hz. Reference electrodes were placed on the left ear lobe.

EEG preprocessing was conducted using Python 3.8.5 with MNE-Python (0.20.7). The EEG data were down-sampled to 250 Hz. Next, the EEG data were filtered with a band-pass ranging from 1 to 45 Hz to remove artifacts. Each EEG channel was visually inspected for bad channels. To reduce or eliminate artifacts (electrooculogram, muscle noise, sweating, and movement), we conducted an independent component analysis (ICA) on the EEG. After ICA, we removed independent components derived from artifacts. Bad channels were excluded from the analysis (mean number of bad channels: 2.20, SD: 1.21). The EEG data were band-pass filtered to extract the theta (4–7 Hz), alpha (8–12 Hz), and beta (13–30 Hz) frequency bands.

The inter-brain EEG synchronization was evaluated using CCorr^26,27^ and PLV^27^ for each electrode pair between two participants. CCorr was directly parallel to the Pearson product-moment correlation coefficient for the circular data.

7 $$\begin{array}{c}CCorr=\left|\frac{\sum_{k=1}^{T} \mathrm{sin}\left({\phi }_{k}-\overline{\phi }\right)\mathrm{sin}\left({\psi }_{k}-\overline{\psi }\right)}{\sqrt{\sum_{k=1}^{T} {\mathrm{sin}}^{2}\left({\phi }_{k}-\overline{\phi }\right){\mathrm{sin}}^{2}\left({\psi }_{k}-\overline{\psi }\right)}}\right|\end{array}$$

where ${\phi }_{k}$ and ${\psi }_{k}$ are the k^th^ EEG phases of participants A and B, extracted using the Hilbert transform from theta, alpha, and beta frequency band passed signals, respectively. $\overline{\upphi }$ and $\overline{\uppsi }$ are the average T phases for EEG channels of participant A and B, respectively. $k$ is the time point of down sampled EEG (250 Hz), and T is the number of EEG time points of participants A and B of the corresponding trial. The CCorr values ranged from 0 to + 1. Afterwards, the calculated CCorr values were normalized by Fisher’s Z transformation.

PLV focuses on the phase relationship between two signals^28^ and is calculated as below:

8 $$\begin{array}{c}PLV=\frac{1}{T}\sum_{k=1}^{T}{e}^{j\left({\varphi }_{x}-{\varphi }_{y}\right)}\end{array}$$

where ${\varphi }_{x}$, ${\varphi }_{y}$ are the phase angle of signals x and y. These phase angles were extracted using Hilbert Transform. k is the time point of sampled EEG, and T is the total number of EEG time points. If PLV = 1, phases are perfectly synchronized in a specific frequency, and if PLV = 0, they are unsynchronized. The *j* is the complex number.

To detect the region of inter-brain synchronization related to the stability of anti-phase tapping, we created the six ROIs for the EEG electrodes (Fig. 4). We calculated the average measures (CCorrs or PLVs) for each ROI pair as follows: (1) we calculated the inter-brain measures over every possible combination of inter-brain EEG channels (total of 841); (2) we averaged the measures included in each ROI pair. For example, for the occipital-occipital ROI pair, measures of four channel pairs, i.e., O1-O1, O1-O2, O2-O1, and O2-O2, were averaged. In addition, the measures were averaged across symmetrical ROI pairs (e.g., for the right temporal-occipital ROI pair, measures of the 12 cannel pairs, i.e., O1-T8, T8-O1, O1-CP6, CP6-O1, O1-P8, P8-O1, O2-T8, T8-O2, O2-CP6, CP6-O2, O2-P8, and P8-O2 were averaged) because there was no distinction between the two participants of a pair, resulting in 21 ROIs pairs (see Fig. 4 for the number of channel pairs averaged in each ROI pair).

### Statistical analyses

#### Behavioral analysis

We performed one-way repeated ANOVA was conducted to determine the effects of tapping condition on SDRP. To confirm the equality of variances of the differences among levels, sphericity was examined using Mauchly’s test. The sphericity of ANOVA with repeated measures (within-subject factors) confirms the equality of variances of the differences among levels. Sphericity is a necessary and sufficient condition for conducting ANOVA^45^.

#### EEG analysis

We conducted Wilcoxon signed rank (paired nonparametric test)^31^ to compare inter-brain EEG synchronization between pseudo tapping condition and slow, fast, and free tapping conditions, respectively. The multiple comparisons were corrected by the Benjamini–Hochberg method.

Correlation between SDRP and inter-brain EEG synchronization: We used Spearman’s correlation. Only ROI pairs that were significantly larger than pseudo tapping were included in the correlation analysis. These p-values of the correlations were corrected by the Benjamini–Hochberg method.

Statistical analyses were conducted using Scipy (https://scipy.org/) and JASP (https://jasp-stats.org/).

## Supplementary Information

Supplementary Information.

## Data Availability

The datasets generated and analyzed during the current study are available from the corresponding author on reasonable request.
