# Supplementary material for: The relationship between stability of interpersonal coordination and inter-brain EEG synchronization during anti-phase tapping
Source: Sci Rep. 2022 Apr 13;12:6164. doi: 10.1038/s41598-022-10049-7 (PMC9008014; doi:10.1038/s41598-022-10049-7)
Supplement: Supplementary file 1 — Supplementary Information. [file 41598_2022_10049_MOESM1_ESM.pdf]

Supplementary Information

The Relationship between Stability of Interpersonal Coordination and Inter-Brain EEG Synchronization during Anti-phase Tapping

Yuto Kurihara, Toru Takahashi, Rieko Osu*

*Correspondence to: r.osu@waseda.jp

1. Results obtained by Coherence

1.1 Comparison to pseudo tapping

We used SciPy (1.7.1) in Python (3.8.5) to calculate coherence. Significant inter-brain synchronizations were observed in multiple ROI pairs in the fast condition in beta band, compared to the pseudo condition (Figure S1). Alternatively, there was no significant ROI synchronization in the theta or alpha bands in the fast condition, nor in any frequency band in the slow or free conditions compared to the pseudo condition.

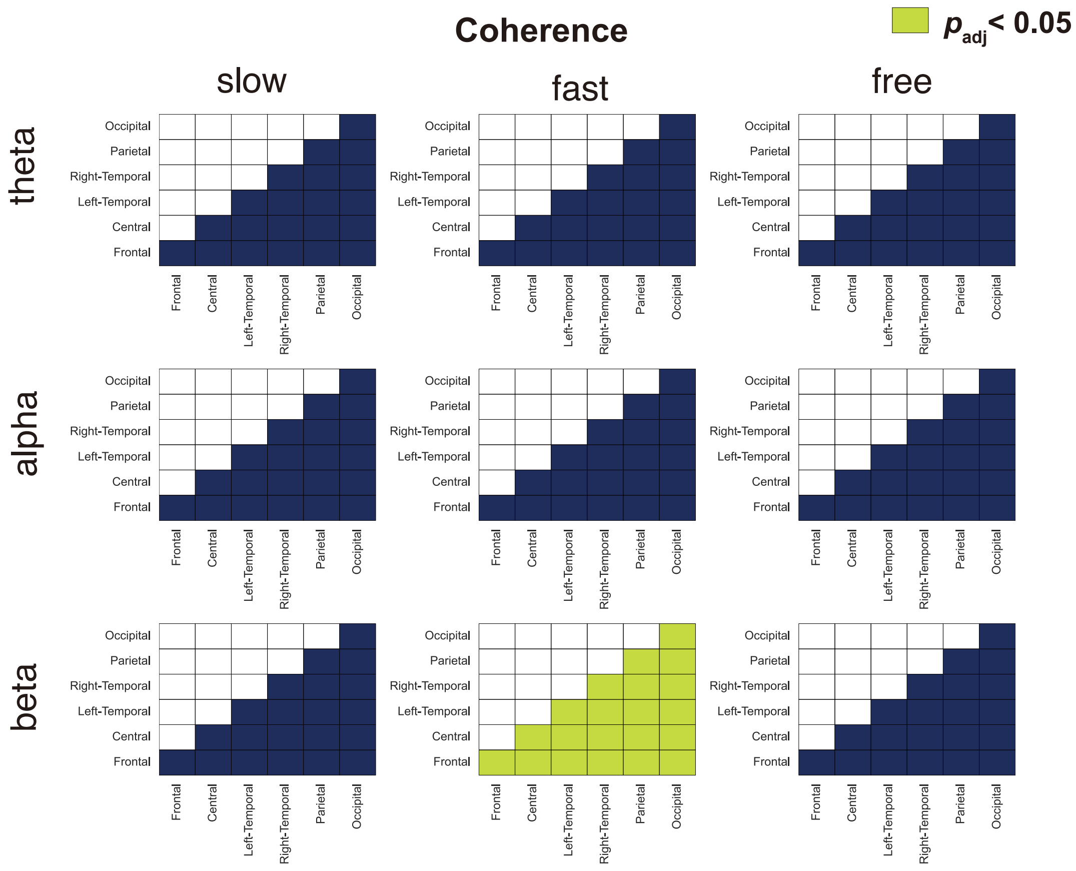

Figure S1: Comparison between coherence of pseudo condition and coherence of slow, fast, and free conditions in theta, alpha, and beta frequency bands. The matrices contain the p-value of Wilcoxon signed rank test comparing coherences during interactive tapping (slow, fast, and free conditions) and pseudo tapping in each pair of regions of interest. ROI pairs that had significantly larger coherences between participants during anti-phase tapping compared to those during pseudo tapping are indicated by red in the corresponding cells with p_{adj} <0.05.

1.2 Correlation with SDRP

There was significant correlation between SDRP and the strength of inter-brain synchronization between central and frontal ROIs for beta band in the fast condition (Figure S2).

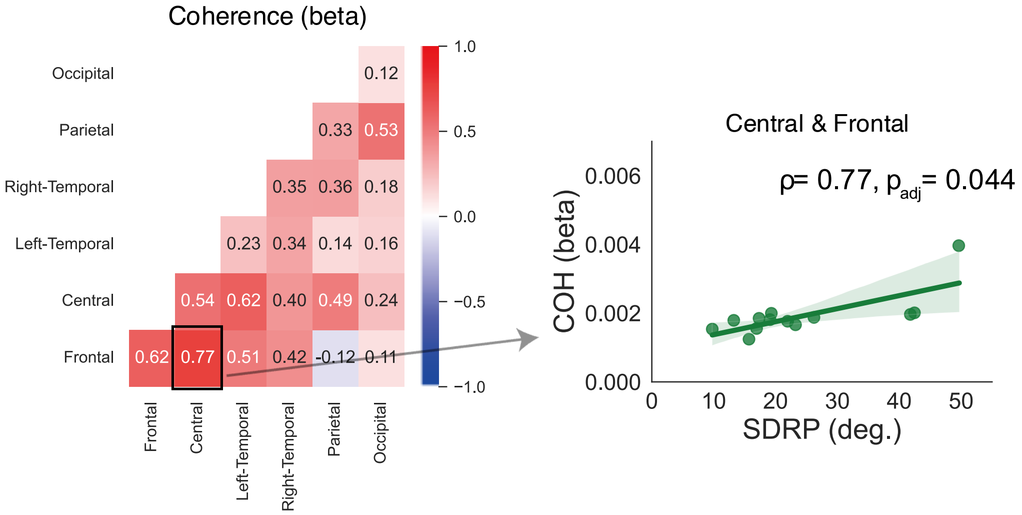

Figure S2: Relationship between beta band coherence and SDRP in the fast condition. The heatmap indicates Spearman's correlations between SDRP and coherence in the beta frequency band.

2. Comparison to surrogate

2.1 Creation of a surrogate dataset

We tested with surrogate data as a control to examine whether the conclusions of this study are robust against methods by which the control data were obtained. The surrogate data were created as follows: 1) we created 1000 ms windows of one participant's EEG time series in each pair of channels in slow, fast, free, and pseudo conditions and shuffled the order of time series (1000 ms window) across four conditions; 2) we extracted the 156 s (average time for tapping in four tapping conditions) windows from the shuffled EEG data; 3) we calculated inter-brain synchronization indices (CCorr/PLV/Coherence) from the surrogate data for each pair of EEG channels; and 4) we averaged the inter-brain synchronization indices within each ROI pair. We conducted these four steps 50 times and created 50 averaged indices (CCorr/PLV/Coherence) for each ROI pair. These steps were applied for the theta, alpha, and beta frequency bands, respectively.

2.2 Comparison of CCorrs, PLVs and coherences between surrogate and original data

To examine whether the observed EEG synchronization was obtained by chance, we compared the indices between original and surrogate data for 21 ROI pairs by Wilcoxon signed rank test (one-tail)

with false discovery rate (FDR) correction (number of comparisons, 21 ROI pairs) for each speed condition and frequency band.

Figure S3 shows the CCorr results. Among CCorrs of 21 ROI pairs, we detected 11 significantly larger CCorrs than surrogate CCorrs in the alpha bands and 10 significantly larger CCorrs than surrogate CCorrs in the beta bands in the fast condition. No CCorr was significantly larger in the other condition or other frequency. We calculated Spearman's correlation between SDRP and average CCorrs of the ROI pairs that were significantly correlated with the alpha CCorr between the right temporal and central ROIs (Figure S3A). There was also significant positive correlation between the SDRP and CCorrs in frontal-frontal ROI pairs in the beta bands (Figure S3B). In these regions, the inter-brain synchronization increased when the tapping was less stable.

Figure S4 shows the PLV results. Among PLVs of 21 ROI pairs, we observed 13 significantly larger PLVs than surrogate PLVs in the alpha bands and 17 significantly larger PLVs than surrogate PLVs in the beta bands in the fast condition. There were no ROI pairs which were significantly larger PLVs than surrogate PLVs in the alpha bands. Next, we calculated Spearman's correlation between SDRP and average PLVs of the ROI pairs that were significantly larger than surrogates in the fast condition. There were no significant correlation in the alpha and beta bands (Figure S4A, S4B).

Figure S5 shows the coherence results. Among coherences of 21 ROI pairs, we found 13 significantly larger coherences than surrogate coherences in the theta bands and 17 significantly larger coherences than surrogate coherences in the beta bands in the fast condition. No coherence was significantly larger in the other condition or other frequency. Next, we calculated Spearman's correlation between SDRP and coherence in the ROI pairs that were significantly larger than surrogates in the fast condition. There was a significant positive correlation between SDRP and alpha PLV in the central and frontal ROI pairs (Figure S5B). However, there were no significant correlation in the theta bands (Figure S5A).

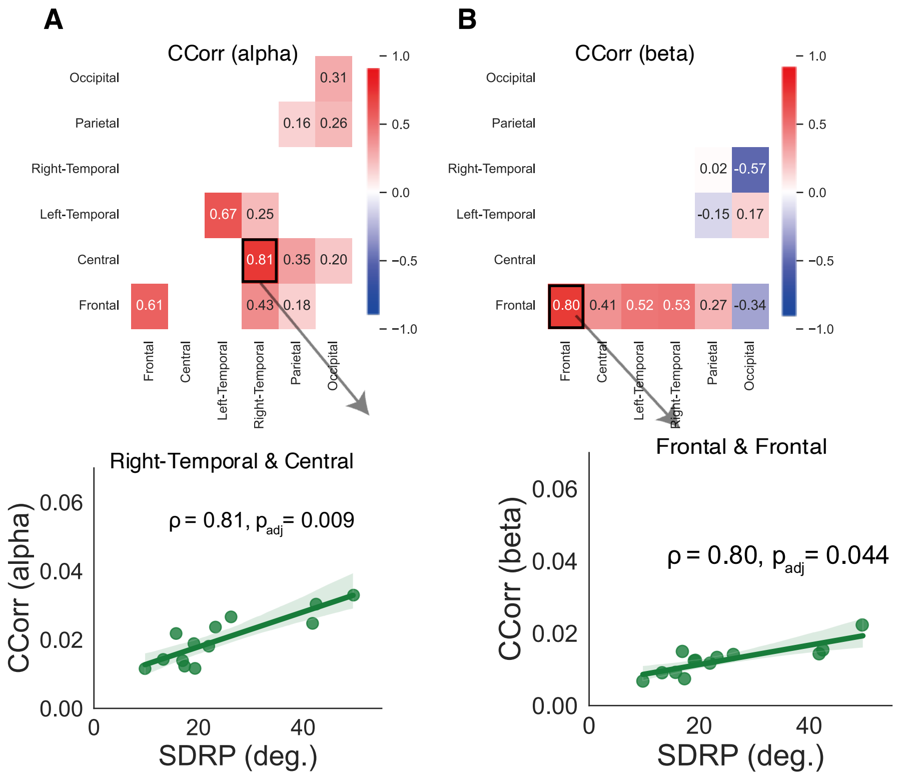

Figure S3: Relationship between inter-brain CCorr and the instability of anti-phase tapping in the fast condition using surrogate method. (A) The heatmap indicates Spearman's correlations between SDRP and CCorr in the alpha frequency band. The scatter plot shows a significant positive correlation between SDRP and CCorr. (B) The heatmap indicates Spearman's correlations between SDRP and CCorr in the beta frequency band. The scatter plot shows a significant positive correlation.

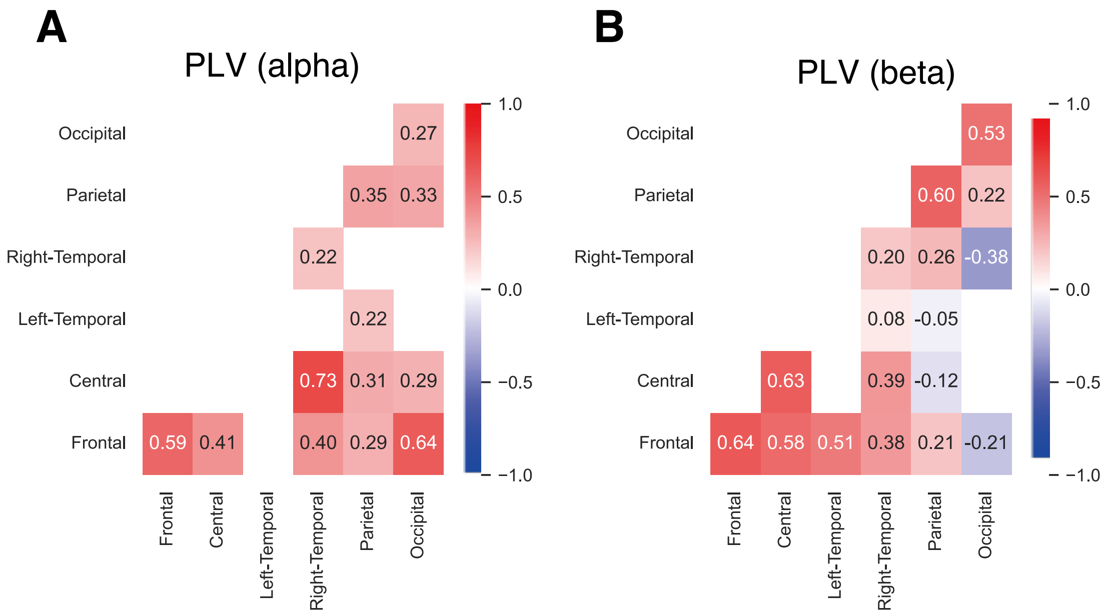

Figure S4: Relationship between inter-brain PLV and the instability of anti-phase tapping in the fast condition using surrogate method. (A) The heatmap indicates Spearman's correlations between SDRP and PLV in the alpha frequency band. (B) The heatmap indicates Spearman's correlations between SDRP and PLV in the beta frequency band.

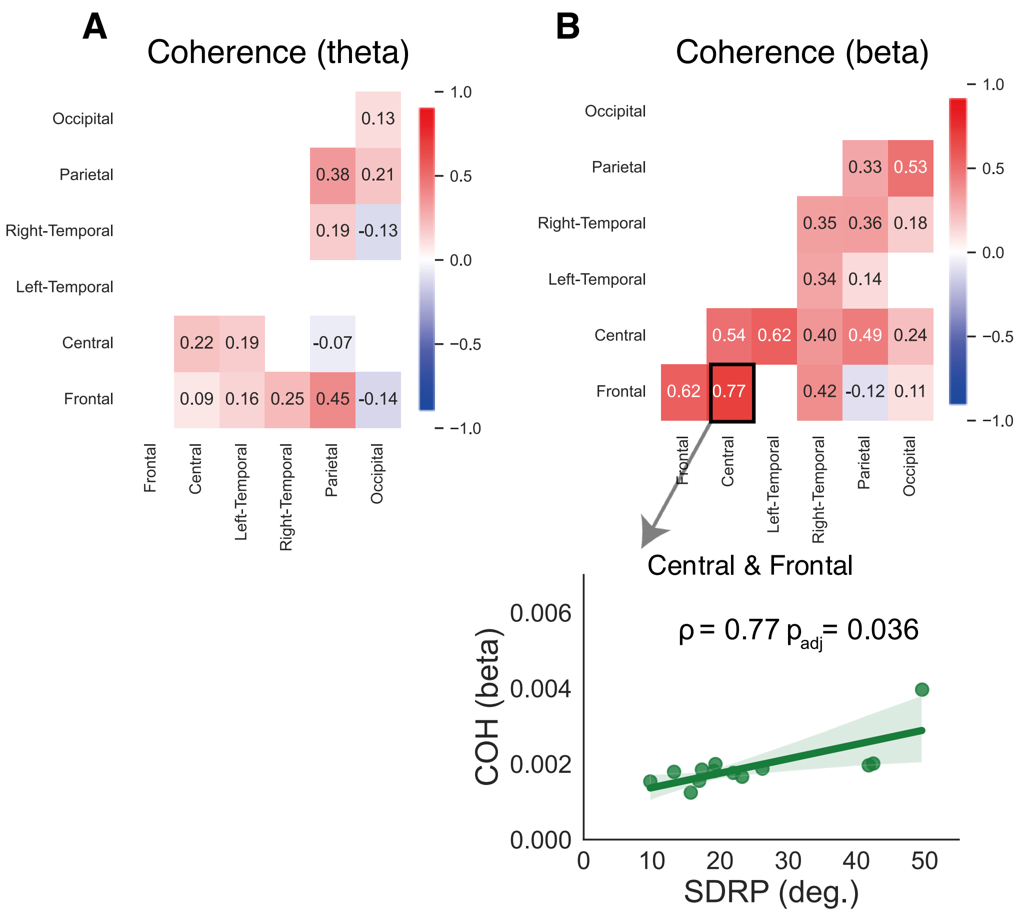

Figure S5: Relationship between inter-brain coherence and the instability of anti-phase tapping in the fast condition using surrogate method. (A) The heatmap indicates Spearman's correlations between SDRP and coherence in the theta frequency band. No correlations were significant. (B) The heatmap indicates Spearman's correlations between SDRP and coherence in the alpha frequency band. No correlations were significant. (C) The heatmap indicates Spearman's correlations between SDRP and coherence in the beta frequency band. The scatter plot shows a significant positive correlation between SDRP and coherence between the central and the frontal region.

3. Low, Middle, and High Beta frequency bands

Beta EEG oscillation is conventionally split into three sections: low (beta1: 13–16 Hz); middle (beta2: 16.5–20 Hz); and high (beta3: 20.5–28 Hz). Therefore, we reanalyzed inter-brain EEG synchronization separately for beta1, beta2, and beta3. The difference in ROI-averaged CCorr/PLV between interactive tapping and pseudo tapping was tested by one-tail Wilcoxon signed rank test with false discovery rate (FDR) correction (number of comparisons was 21 ROI pairs) for each tapping condition (slow, fast, and free) in beta1, beta2, and beta3. We observed 19 significantly larger CCorrs in the beta1 bands, three significantly larger CCorrs in beta2, and eight significantly larger CCorrs in beta3 in the fast condition (Figure S6). We observed 19 significantly larger PLVs in the beta1 bands, nine significantly larger PLVs in beta2, and 10 significantly larger PLVs in beta3 in the fast condition (Figure S7). Furthermore, we calculated the Spearman's correlation between SDRP and ROI-averaged CCorrs/PLVs that were significantly larger than pseudo tapping in the fast condition. There was a significant positive correlation between SDRP and beta3 PLV in central and central ROI pairs (ρ =0.79, p_{adj} = 0.026; Figure S8).

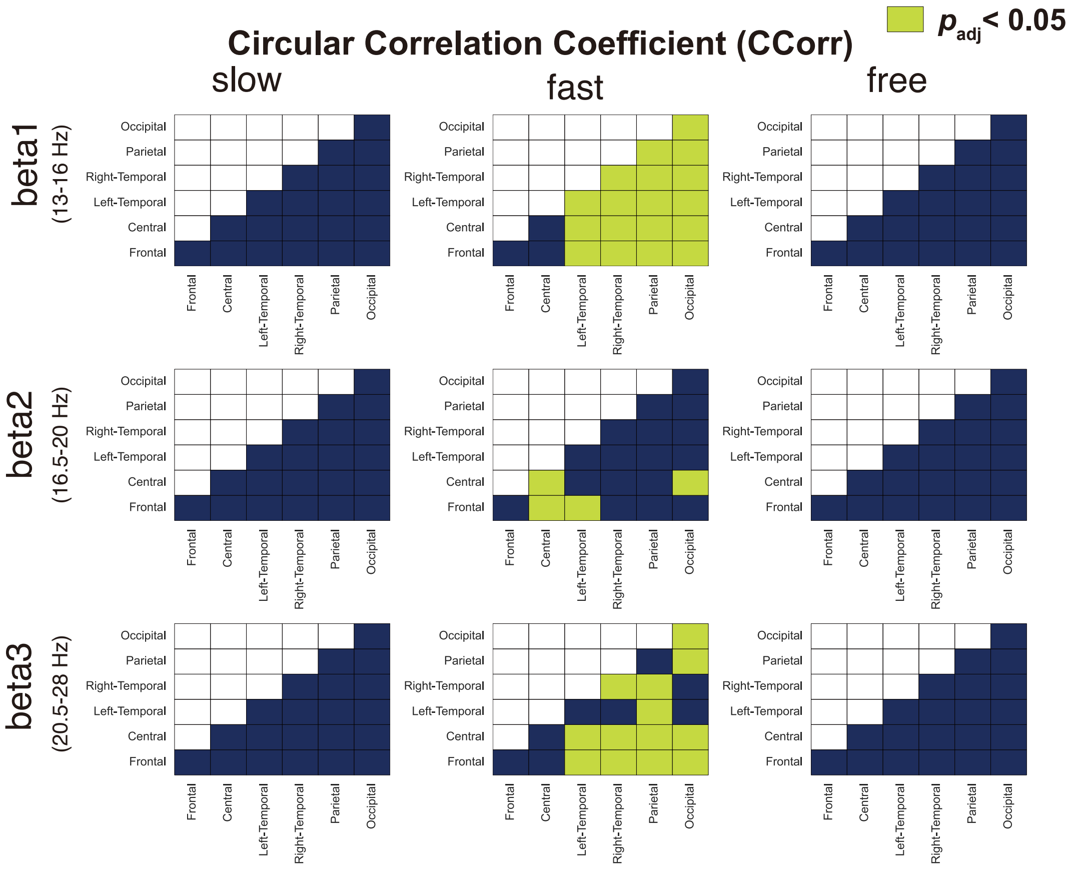

Figure S6: Comparison between CCorr of pseudo condition and Ccorr of slow, fast, and free conditions in beta1, beta2, and beta3 frequency bands.

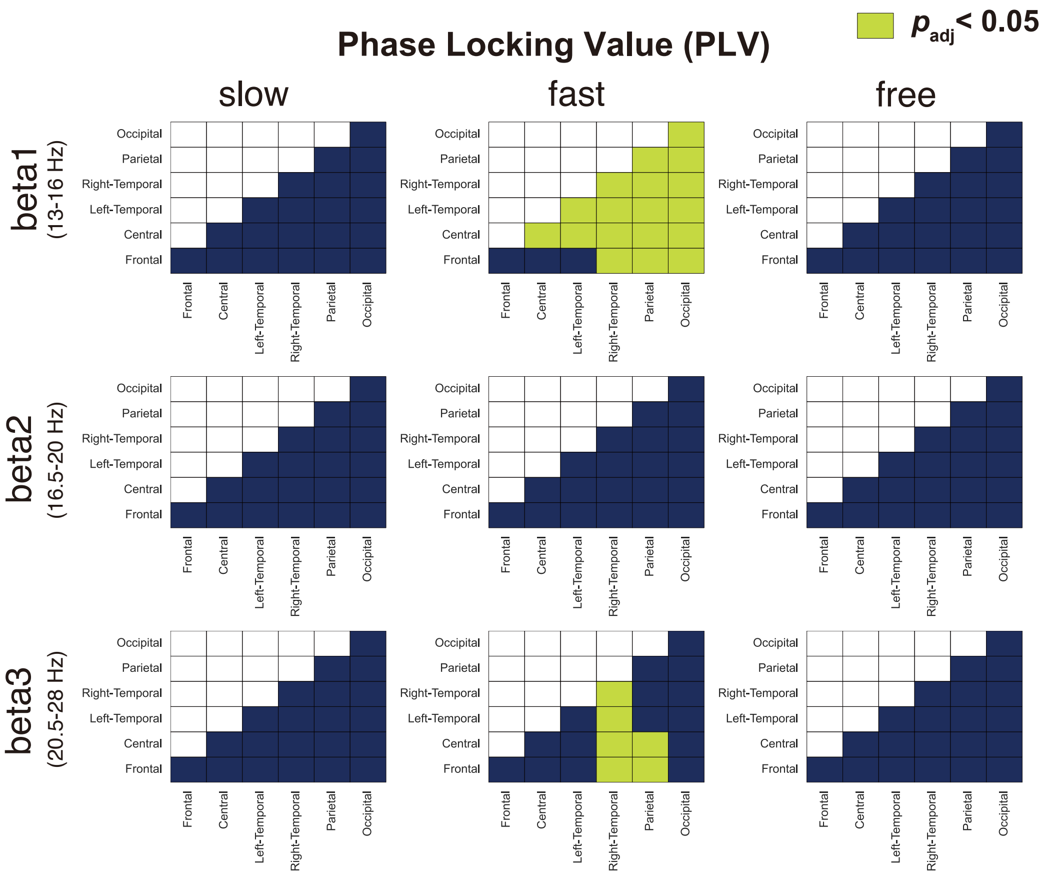

Figure S7: Comparison between PLV of pseudo condition and PLV of slow, fast, and free conditions in beta1, beta2, and beta3 frequency bands.

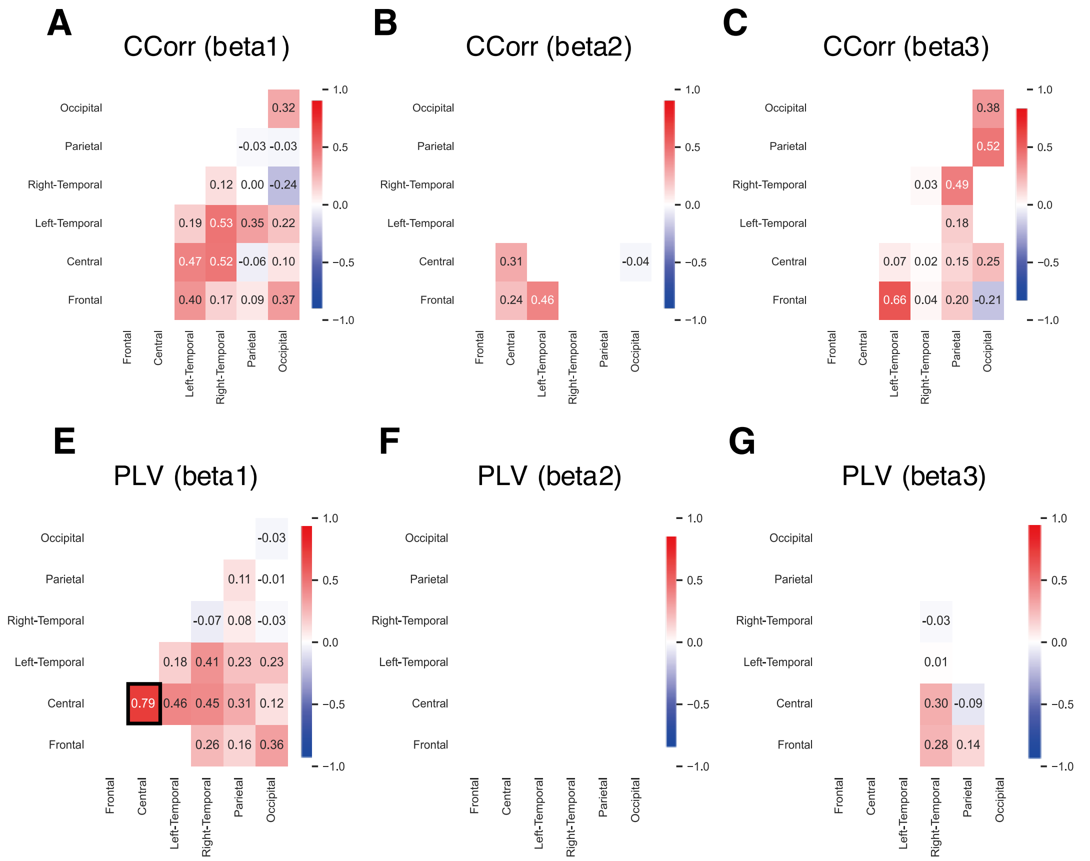

Figure S8: Relationship between inter-brain Ccorr/PLV and the instability of anti-phase tapping in the fast condition. (A) Ccorr of beta1, (B) Ccorr of beta2, (C) Ccorr of beta3, (D) PLV of beta1, (E) PLV of beta2, (F) PLV of beta3. The black enclosure indicates significant correlation between SDRP and inter-brain EEG synchronization.

4. Topographical map in the slow, fast, free, and pseudo condition.

We showed a spectra topographical map by theta, alpha, and beta frequency bands (epoch number=176, window size=0.7s) (Figure S9). The normalized EEG power was averaged within participants. From the Fig. S9, we did not consider that the brain activity was biased to a specific region in all tapping conditions in all frequency bands.

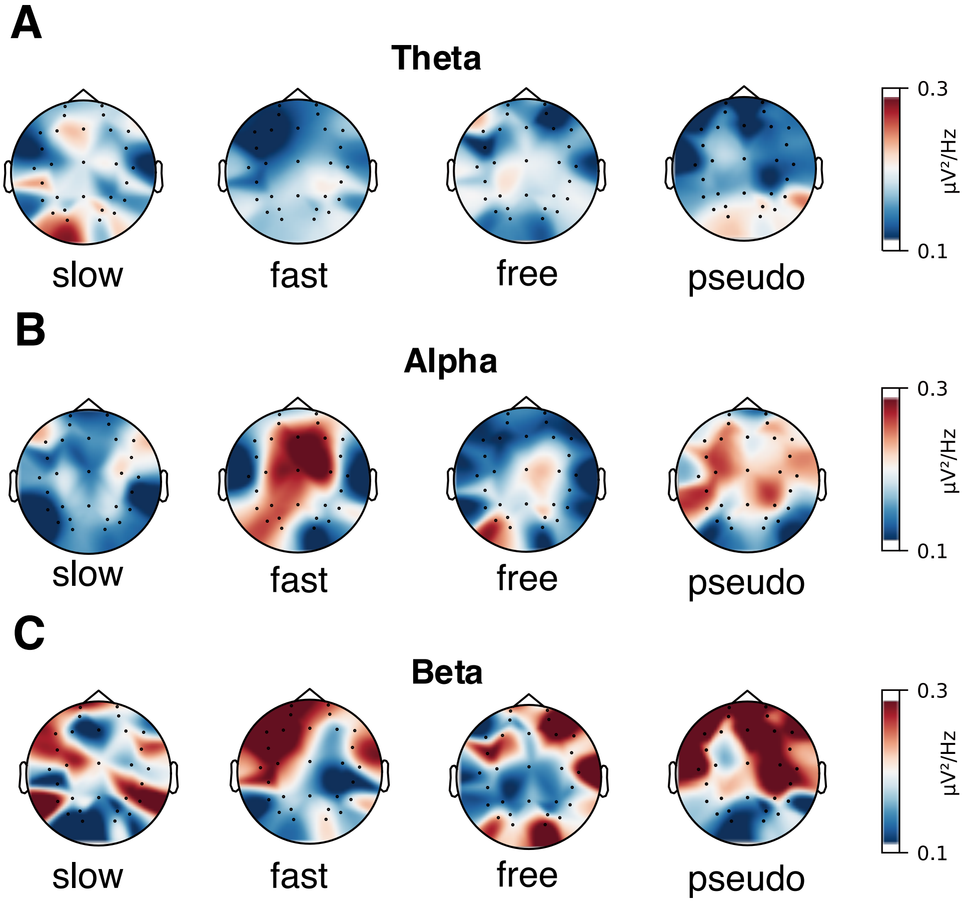

Figure S9: The topographical map in the tapping condition showing the theta, alpha, and beta frequency bands. The normalized EEG power shows the average values of the participants.

5. Channel based Inter-brain connection

We performed a comparison in CCorr/PLV between interactive tapping (slow, fast, and free) and pseudo tapping for each channel pairs using Wilcoxon signed rank test and obtained inter-brain connections which were higher than pseudo ($p < 0.01$ uncorrected). We averaged the CCorrs or PLVs of electrode pairs within each EEG channel pair. The results showed that the fast condition had a larger number of significant inter-brain connections than slow and free condition in theta, alpha, and beta bands ($p < 0.01$) (CCorr: Figure S10, PLV: Figure S11).

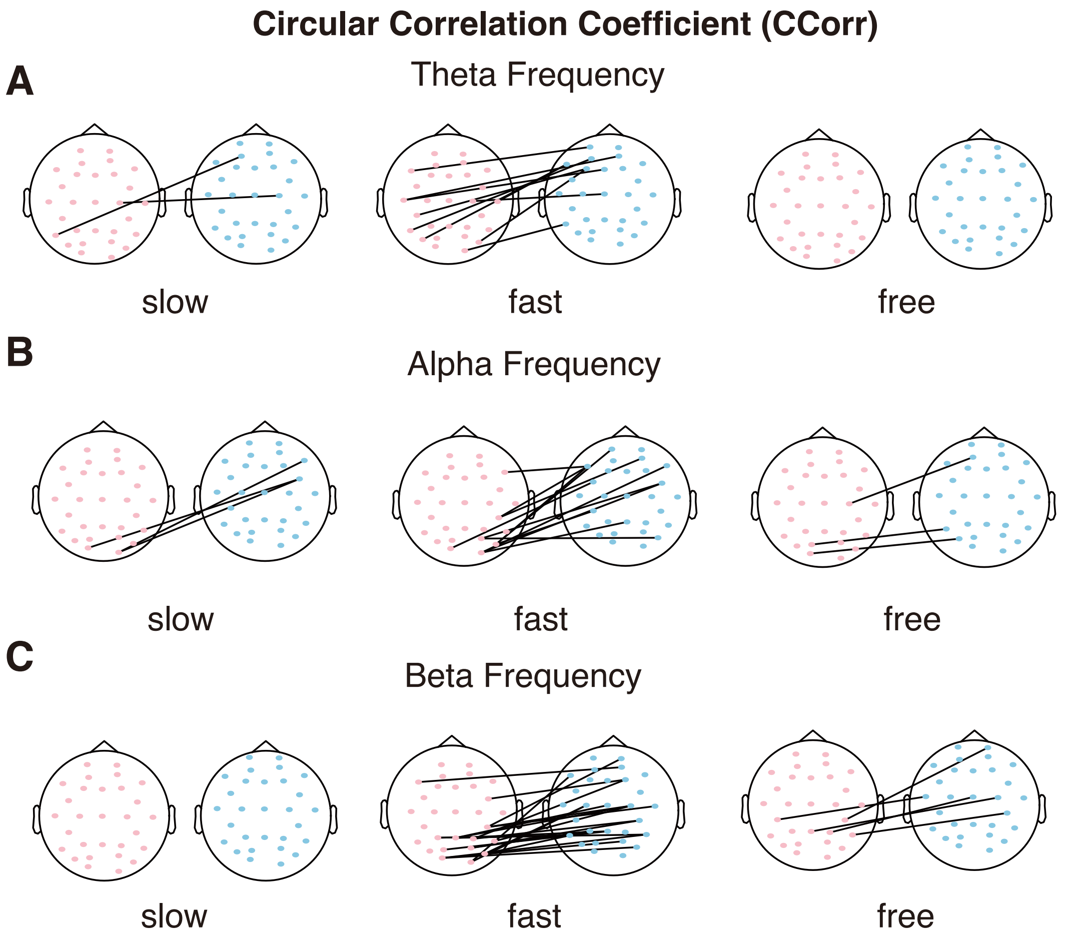

Figure S10: EEG channels pairs showing significantly higher (A) theta, (B) alpha, and (C) beta CCorr between two brains ($p < 0.01$, uncorrected).

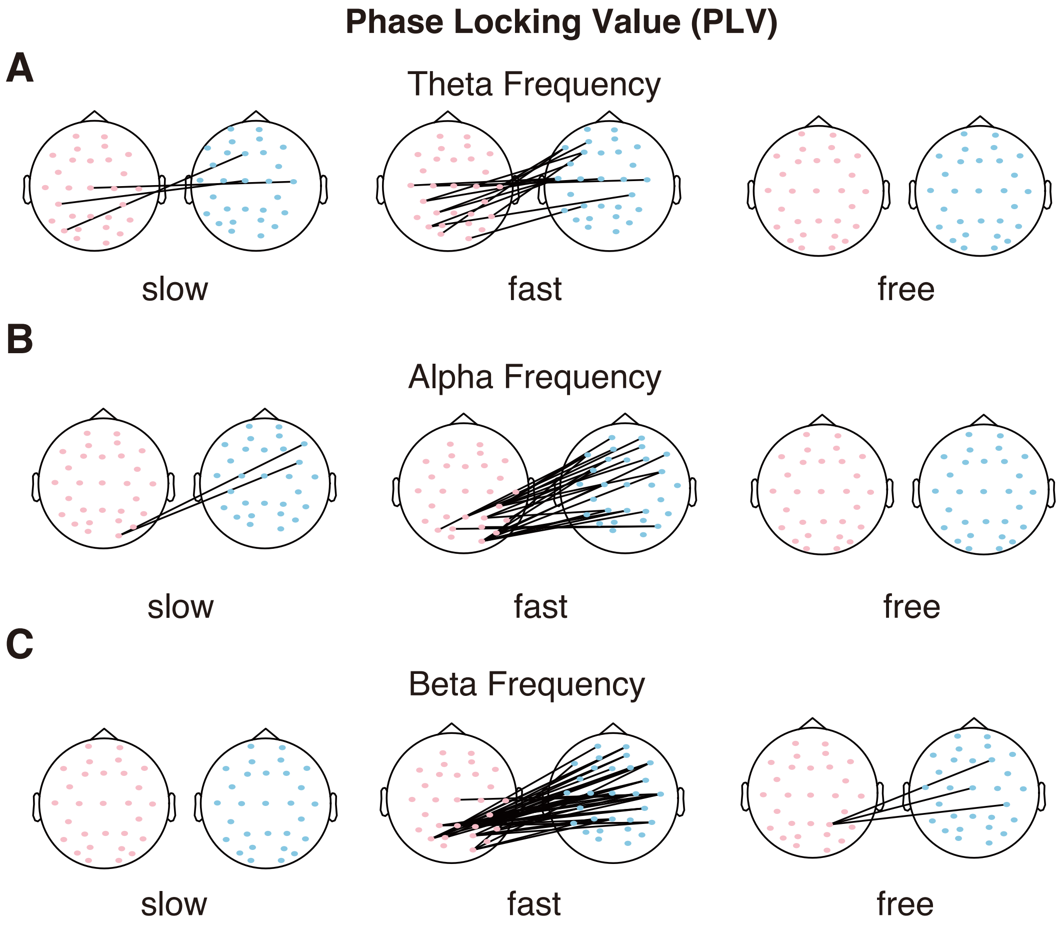

Figure S11: EEG channels pairs showing significantly higher (A) theta, (B) alpha, and (C) beta PLV between two brains ($p < 0.01$, uncorrected).

References

[1]. Mitra, P. P. & Pesaran, B. Analysis of dynamic brain imaging data. Biophys. *J.* 76, 691–708 (1999).

[2]. Lachaux, J. P., Rodriguez, E., Martinerie, J. & Varela, F. J. Measuring phase synchrony in brain signals. Hum. Brain Mapp. 8, 194–208 (1999).

[3]. Lepage, K. Q. & Vijayan, S. The relationship between coherence and the phase-locking value. J. Theor. Biol. 435, 106–109 (2017).

[4]. Srinath, R. & Ray, S. Effect of amplitude correlations on coherence in the local field potential. J. Neurophysiol. 112, 741–751 (2014).
